# Radiology and Electro-Therapeutics in War-Time

**Published:** 1915-05-08

**Authors:** 


### RADIOLOGY AND ELECTRO-THERAPEUTICS IN WAR-TIME.

### Lessons from the Military Hospitals.

In an interesting article in the March issue of the *Practitioner* Dr. Francis Hernaman-Johnson, of the Cambridge Hospital, Aldershot, relates his experiences in dealing with the accidents and emergencies which arise among the new armies preparing for the Front. He notes the rarity of injuries among those who are being taught to deal with horses, and thinks this reflects credit both on the skill of the instructors and the aptitude of the pupils. Football, on the other hand, is responsible for many accidents, and these incapacitate a considerable number of men even for long periods. Fractures of the tibia and of the fibula are the most frequent injuries. They often occur as mere cracks, without any displacement, and can only be diagnosed with certainty by means of skiagrams; in such cases a mere screen examination is valueless.

Many examinations have to be made for suspected calculus of the kidney and bladder. The proportion of positive results is small, but it is noteworthy that hard marches and exposure seem to bring on symptoms suggestive of calculus in a number of men who, previous to such experiences, had appeared to be in good health.

### The Function of Radiography.

In connection with injuries produced in actual warfare the function of radiography is concerned with the diagnosis of bone injuries, and with the localisation of bullets, shell fragments, and other foreign bodies. It may be useful to record some of the lessons which have been learned on these points, especially as there are now many practitioners engaged at military hospitals who have had little experience of such problems in civil practice. With two wounds produced by a bullet or shell fragment the first suggestion is that one of these is an entrance wound and the other the wound of exit—that, in other words, the foreign body has not lodged in the tissues. There is, however, some uncertainty in this conclusion, and in all but the simplest cases a skiagram ought to be taken. One possibility is that the two wounds may have been inflicted by two separate bullets, and even if only one missile is responsible, while part of it may have escaped another part may be retained. Again, serious damage may be inflicted on a bone short of complete fracture. As opposed to all this, the fact of a single wound only does not prove that a foreign body is present in the tissues. A conical bullet may penetrate some distance, and yet may fall out of the wound; or a bullet may have been removed at some earlier stage in the treatment, and the patient be ignorant of this. Again, a bullet may have travelled a considerable distance from the wound which it has inflicted, and hence symptoms of irritation in some remote area or organ call for radiography though an earlier skiagram in the neighbourhood of the wound may have been negative. The mere demonstration of the presence of a foreign body by means of the screen or radiogram is rarely a sufficient guide for its surgical removal; it is necessary also to use a proper localising apparatus, for without this an attempt to remove the foreign body is likely to fail.

In the department of electro-therapeutics help is afforded in the following directions: the gravity or otherwise of injuries to nerves and muscles can be determined; adhesions may be softened and absorbed; recovery from paralysis can be hastened; while by the use of radiant heat, high frequency currents, and so on such conditions as rheumatism, neuritis, and fibrositis can be successfully treated. For the electrical testing of muscles and nerves the best apparatus is a Lewis Jones condenser set, by which discharges varying from 1-2,000th to 1-40th of a second can be obtained. Muscles responding only to the longest discharges are certainly in a state of degeneration. On the other hand, in cases of "psychical" paralysis a response is obtained to the slightest condenser discharges. In some of these latter cases, however, there is some degree of failure in response, and therefore an indication that a measure of organic change exists. These cases are commonly spoken of as "hysterical," but they are certainly not examples of malingering; probably if our methods of investigation were sufficiently delicate evidences of a physical basis for the paralysis would be obtained in all of them.

### The Physician's Point of View.

In cases of nerve lesions of moderate severity the effect of ionisation combined with faradisation of the affected muscles is little short of marvellous; the treatment, if properly applied, can return to the fighting line in the course of a few weeks thousands of men who otherwise would have required months to recover. Even when a complete cure cannot be obtained great benefit can be secured; and though the patient may not gain the activity needed by the military authorities, he may be made capable of work in civil life. In present circumstances treatment of these latter can hardly be undertaken in the military hospitals, which have to concentrate themselves on patients who may with reasonable promptness be fitted for active service. From the physician's point of view, however, such cases demand assistance, and it is much to be desired that modern methods of treatment will be secured for them. Failing this, they will surely fall into the hands of quacks and charlatans.

The subject is not mentioned in the paper here summarised, but there is increasing evidence that the efficient application of ionisation is able to exercise a most beneficial influence on the healing of wounds and sinuses which have proved refractory to the more ordinary methods. Some of the officers who have recently joined the R.A.M.C. have experience of this form of treatment, and it may be expected that they will get opportunities of putting the treatment into operation.

